# Compassionate use of others’ immunity — understanding gut microbiome in Covid-19

**DOI:** 10.1186/s13054-020-03043-w

**Published:** 2020-06-18

**Authors:** Prashanth Thalanayar Muthukrishnan, Robert Faillace

**Affiliations:** 1 Pulmonary and Critical Care Medicine, Department of Medicine, Albert Einstein College of Medicine, NYC HHC, Jacobi Medical Center and North Central Bronx Hospital, 1400 Pelham Parkway S, Bronx, NY 10461 USA; 2 Department of Medicine, Jacobi Medical Center, Albert Einstein College of Medicine, NYC HHC, 1400 Pelham Parkway S, Bronx, NY 10461 USA

Disease is a complex interaction between disease-causing agent, host, and the environment. About 90–95% of the public that test positive for coronavirus recover; hence, most of us have inbuilt host response that creates a life-sustaining relationship with coronavirus. Shen and colleagues have used plasma from survivors to study the amplification of host response in severe Covid-19 patients [1]. Modern medicine has been documenting the evidence behind the ancient wisdom—“home of immunity is the gut microbiome” [2]. Hence, we address the scope of understanding host response and immunity during this time of global emergency.

About 433 people die every day from lung cancer in the USA, and 1772 people die per day in the USA from heart disease and many from sepsis, too; such chronic diseases continue to be risk factors for Covid-19 mortality. This illustrates the Dean Ornish and Ayurvedic connection between dysregulated lifestyle, microbiome diversity, chronic disease, and morbidity from communicable and non-communicable disease pandemics. The dysregulation of functional (vpk) forces in the gut apparatus that arise from epigenetic influences accumulates over time, initially asymptomatic in young age and later manifests as hypertension, heart disease, diabetes, migraines, asthma, cancer, and susceptibility to morbidity from infection/dysregulated inflammatory response (Sepsis, etc.) [2].

In our search for an immediate fix to this immune dysregulation in 5–10% of the world population that is critically ill, we are trying to understand the characteristics of the immunity (vpk phenotype assessment) of people who tested positive and survived with good prognosis. Per Brandt, fecal transplants have helped in severe C.diff infections by restoring the gut integrity (aka gut microbiome immunity) from other healthy individuals [3]. There is literature on the experimental use of fecal transplant in sepsis and multi-organ dysfunction syndrome with and without C.diff infection by Wei et al. from China [4, 5]. Our team is considering stool microbiome studies and the role of transplant from healthy donors and milder Covid-positive patients as a compassionate use experimental treatment for critically ill patients. Precautions are necessary because we know that Covid-19 virus is shed in the stool samples of Covid-positive patients. We invite inputs from colleagues on benefit:risk assessment of Covid-19 stool microbiome studies and anecdotal use of FMT such as by Wei et al.

In summary, in the same lines as using convalescent sera from milder Covid patients as a therapy, we are enquiring into potential ongoing considerations for studying the microbiome in Covid-19 as a diagnostic/therapeutic tool.

## Data Availability

This is a letter to the editor. Data supporting the content of the letter is present as previous literature quoted as references in the manuscript.
